# Physiological, Nutritional and Transcriptomic Responses of Sturgeon (*Acipenser schrenckii*) to Complete Substitution of Fishmeal with Cottonseed Protein Concentrate in Aquafeed

**DOI:** 10.3390/biology12040490

**Published:** 2023-03-23

**Authors:** Chang’an Wang, Zhigang Zhao, Shaoxia Lu, Yang Liu, Shicheng Han, Haibo Jiang, Yuhong Yang, Hongbai Liu

**Affiliations:** 1 Key Laboratory of Aquatic Animal Diseases and Immune Technology of Heilongjiang Province, Heilongjiang River Fisheries Research Institute, Chinese Academy of Fishery Sciences, Harbin 150070, China; 2 College of Animal Science and Technology, Northeast Agricultural University, Harbin 150030, China; 3 College of Animal Science, Guizhou University, Guiyang 550025, China

**Keywords:** *Acipenser schrenckii*, liver, transcriptome, differentially expressed genes

## Abstract

**Simple Summary:**

Cottonseed protein concentrate (CPC) is a high-protein product derived from widely available cottonseed flakes, but its use is limited by the presence of anti-nutritional components (gossypol, etc.). This study investigated the potential of using CPC as a substitute for fishmeal in the diet of sturgeon (*Acipenser schrenckii*). The study found that completely replacing fishmeal with CPC had negative effects on the growth and physiology of the sturgeon, as shown by reduced weight gain, feed efficiency, and essential amino acids. Additionally, there was a decrease in digestive enzyme activity and an upregulation of genes linked to metabolism in the liver. The research concluded that CPC should not completely replace fishmeal in the diet of the sturgeon. This study provides important data for the development of better aquafeeds and the evaluation of diet performance in sturgeon using molecular methods. Although plant proteins such as CPC can reduce production costs in aquaculture, their excessive consumption can lead to the loss of growth performance and digestion due to anti-nutritional components.

**Abstract:**

This study estimated the effect of substituting fishmeal completely with cottonseed protein concentrate (CPC) in the diet of sturgeon (*Acipenser schrenckii*) on growth, digestive physiology, and hepatic gene expression. A control diet containing fishmeal and an experimental diet based on CPC was designed. The study was conducted for 56 days in indoor recirculating aquaculture systems. The results showed that weight gain, feed efficiency, and whole-body essential amino acids (EAAs) all decreased significantly in the experimental group, while whole-body non-essential amino acids (NEAAs) and serum transaminase activity increased (*p* < 0.05). The activity of digestive enzymes in the mid-intestine was significantly reduced (*p* < 0.05), and liver histology revealed fatty infiltration of hepatocytes. The hepatic transcriptome revealed an upregulation of genes linked to metabolism, including steroid biosynthesis, pyruvate metabolism, fatty acid metabolism, and amino acid biosynthesis. These findings indicate that fully replacing fishmeal with CPC harms *A. schrenckii* growth and physiology. This study provides valuable data for the development of improved aquafeeds and the use of molecular methods to evaluate the diet performance of sturgeon.

## 1. Introduction

The use of fishmeal in aquaculture animal diets has been a hot topic in aquatic animal nutrition and feed research. Studies have shown that fishmeal, a source of marine animal protein, is irreplaceable or exceptional in aquatic animal diets [1]. However, declining fishmeal supplies and increasing prices have prompted research into alternatives to fishmeal protein in aquafeeds [2]. Plant proteins are more widely accessible, less expensive, and simpler to produce, making them a good option to replace fishmeal. Currently, many plant proteins are used in aquaculture diets, such as soybean protein concentrate [3], cottonseed meal [4], peanut meal [5], fermented soybean meal [6], and rapeseed meal [7]. Studies have also explored the effect of plant proteins on the development and physiological function of aquatic animals [8], and the results show that optimum levels of plant protein improve growth performance and immunity, while excessive consumption may cause a loss of growth performance and immunity due to anti-nutritional components including phytic acid, protease inhibitors, free cotton phenols, and lectins [9,10,11].

Cottonseed protein concentrate (CPC), a high-protein product derived from cottonseed flakes, has received attention due to its large-scale availability [12]. China and India are the two major cotton-producing countries, producing twice as much as the United States [13]. Although CPC is less cost-effective per unit of protein compared to fishmeal and soybean meal [14], it has the potential to reduce aquaculture production costs significantly if used correctly. To produce CPC, cottonseed flakes are subjected to aqueous alcohol extraction to diminish the content of soluble carbohydrates and anti-nutritional factors [12]. Compared to other alternative protein sources such as canola, rapeseed, sunflower, linseed, lupine, soybean, and corn gluten, less research has been conducted on the substitution of CPC for fishmeal in aquaculture [15]. One of the main limiting factors in using CPC is the presence of gossypol (C_30_H_30_O_8_), a yellow polyphenolic molecule composed of reactive aldehydes and hydroxyl groups, which is the principal anti-nutritional component in CPC that can harm fish [16]. Therefore, to ensure normal fish development, the ratio of CPC to fishmeal in the diet should not be too high.

Currently, research has focused on identifying optimal levels of substitution of fishmeal with other ingredients for different species of fish, including catfish (*Pseudobagrus ussuriensis*) [16], black sea bass (*Centropristis striata*) [14], black sea bream (*Acanthopagrus schlegelii*) [17], Nile tilapia (*Oreochromis niloticus*) [18], and turbot (*Scophthalmus maximus*) [19]. Previous studies have discovered that CPC can substitute 20% to 50% of fishmeal protein without negatively impacting aquatic animal development. However, substituting 36% of the fishmeal in the diet of golden pompano (*Trachinotus ovatus*) with CPC resulted in considerable losses in whole-body crude protein [12], while replacing 50% of the fishmeal resulted in a significant weight reduction in red drum (*Sciaenops ocellatus*) [20]. The mechanism behind such adverse consequences is not completely understood [21,22]. It was revealed that substituting 45% of fishmeal in diets with CPC increased gossypol remnants [17,22] and caused intestinal inflammation [23], but their link to the negative effects of CPC replacement is unknown.

High-throughput mRNA sequencing (RNA-Seq) is a commonly used technique to uncover gene expression differences in non-model animals without reference genome data [24]. Moreover, several studies have been carried out in order to investigate the differential gene expression in metabolism pathways through transcriptomic analysis in various fish species, for instance, rainbow trout (*Oncorhynchus mykiss*) [25], Atlantic salmon (*Salmo salar*) [26,27], and grass carp (*Ctenopharyngodon idella*) [28]. The liver is very important in metabolism because it performs a variety of functions, including gluconeogenesis, storage, and regulation of nutrients, and detoxification of harmful substances. Additionally, it is involved in the regulation of bile secretion. However, the effect of a fishmeal-free diet on the metabolism process in fish livers remains unclear. Thus, the liver transcriptomic analysis may help in better understanding the metabolic processes induced by dietary feed stuff augmentation in aquaculture.

Amur sturgeon (*Acipenser schrenckii*), a native freshwater fish indigenous to the Amur River basin of China and Russia [29], has remarkable evolutionary, economic, and conservation value and is one of China’s most widely farmed sturgeons [30]. Despite its popularity, there is limited research on its dietary requirements and the utilization of plant protein in its diet. This study aims to use RNA-Seq to uncover the molecular mechanism behind the transcriptome profile of the liver of *A. schrenckii* when fed a CPC-based fishmeal-free diet. Additionally, its effects on growth, body composition, and physiological and metabolic responses will be analyzed to evaluate the efficiency of nutrient delivery and utilization of CPC in the *A. schrenckii* diet. This study will give useful information about the physiological and metabolic effects of feeding sturgeons a CPC-based fishmeal-free diet and contribute to the development of improved nutrient supply and CPC utilization techniques in aquatic animal diets.

## 2. Materials and Methods

### 2.1. Experimental Diets

The control group used 50% fishmeal as the main protein component, while the treatment group used 50% cottonseed protein concentrate (CPC). Balancing the nutritional content of the diet by substituting fish oil and crystalline amino acids for gross energy and essential amino acids (EAAs). To adjust the overall phosphorus level of the test diet, calcium dihydrogen phosphate, Ca(H_2_PO_4_)_2_, was added. The trace amount of 0.1% yttrium oxide (Y_2_O_3_) was supplemented into diets as an inert marker to measure the digestibility. Table 1 displays the diet formulation and composition.

In the preparation of the feed, the ingredients were ground to a particle size of 250 μm using a laboratory grinder. Afterward, these ground ingredients were blended with additional components, including micronutrients such as vitamins and minerals. The dry mix was stirred for 15 min to ensure homogeneity. Next, the lipid source and water were added and blended for an additional 10 min using a mixer. The final feed mixture was pelletized into 1.5-mm pellets using the HX-200G pelletizer (Minan Instruments, Jining, China). Diets were then oven dried at 55 °C to reach a moisture content of 8–12%, followed by storage in sealed plastic bags at −20 °C before feeding. The chemical composition of the feed was analyzed using standard techniques, including determinations of moisture, ash, gross energy, crude protein, and crude lipid content [31]. The amino acids in the diets were determined using the techniques described by Fountoulakis and Lahm (1998) [32] and Yust et al. (2004) [33]. High-performance liquid chromatography (HPLC) was employed to measure dietary gossypol concentrations [34].

### 2.2. Farming Management

The Heilongjiang River Fisheries Research Institute Committee for the Welfare and Ethics of Laboratory Animals approved all techniques used in animal experiments (Ethics approval number: 20200615). A total of 180 healthy sturgeons (initial weight: 21.32 ± 0.18 g) were purchased from the Engineering and Technology Center of Sturgeon Breeding and Cultivation (Beijing, China). Sturgeons were randomly allocated to tanks in three replicates, with a density of 30 fish per tank (280 L). After a 14-day acclimatization period in the laboratory setting, sturgeons were given the control diet before the start of the experiment. For 56 days, the sturgeons were raised in an aquatic recirculation system with a constant water flow of around 2.0 L/s. Twice-daily manual feeding was conducted at 9:00 a.m. and 4:00 p.m. until the fish showed signs of satiation. One hour after feeding, any remaining feed was drained from the tank and then oven-dried at 65 °C for 24 h to determine the quantity of feed consumed and its efficiency. According to Stone et al. (2008), feces samples were obtained by hand stripping, which began in line at the front of the pelvic fins and ended at the anus [35]. The feces samples were collected over two weeks to allow for sufficient material for chemical analysis. The trial was conducted under a 12 h dark/12 h light photoperiod. Throughout the trial, daily monitoring of water quality was performed utilizing a YSI 6600 V2-2 instrument (YSI Co., Yellow Springs, OH, USA), and the results indicated dissolved oxygen levels of 7.1–8.3 mg/L, ammoniacal nitrogen of <0.2 mg/L, pH of 7.3–7.8, nitrate of <0.4 mg/L, and nitrite of <0.2 mg/L.

After the trial, sturgeons were starved for 24 h. The weight and total length of each fish were recorded and used to calculate its condition factor (CF). The total weight of sturgeons per tank was aggregated, and the weight growth rate (WGR) and feed conversion rate (FCR) were calculated accordingly.

### 2.3. Sample Collection

Randomly selected sturgeons from each tank were sedated using tricaine methanesulfonate (MS-222, 60 mg/L). Subsequently, blood, whole body, liver, and mid-intestine samples were collected. Four fish were selected from each tank for body composition analysis, amino acid profile analysis, and blood chemical analysis. Blood was obtained from their caudal veins using a syringe containing sodium ethylenediaminetetraacetic acid (EDTA) as an anticoagulant. The collected blood was immediately subjected to centrifugation for 10 min at 7000× *g* to separate the supernatant. Afterward, the serum samples were stored at −20 °C until analyzed. Mid-intestinal samples were obtained from four individuals per tank and preserved at −80 °C for subsequent enzymatic analysis of digestive function. Additionally, livers were selected from six individuals per group for histological examination. Lastly, three individuals in each tank were randomly sampled for transcriptomic analysis of the livers. A rapid extraction procedure was employed to collect liver tissue, which was immediately placed in liquid nitrogen.

### 2.4. Analysis of Body Composition, Apparent Digestibility and Serum Indices

The proximate compositions of diets, feces, and the whole body were analyzed following the protocols provided by Al-Mentafji (2016), which included moisture, crude protein, crude lipid, ash, and gross energy [31]. Moisture content was determined by drying in an oven at 105 °C for 3 h, and crude protein content was analyzed using a Kjeltec machine (2300, Foss Ltd., Höganäs, Sweden). The ether extraction procedure was performed on the Soxtec System (ST-6A, Naai Ltd., Shanghai, China) to determine crude lipid. Ash was determined by muffle furnace incineration at 600 °C for 2 h. The gross energy was measured using a bomb calorimeter (LRY-600A, Chuangxin Ltd., Hebi, China). The yttrium content was quantified using mass spectrometric analysis on the LCMS-8030 system (Shimadzu Ltd., Kyoto, Japan).

The amino acid composition was determined following acid hydrolysis using the Fountoulakis and Lahm technique (1998) [32] on an automated amino acid analyzer (L-8900, Hitachi Ltd., Tokyo, Japan). Tryptophan was hydrolyzed in sodium hydroxide, neutralized, and measured by high-performance liquid chromatography (Dionex, Thermo Fisher Scientific, Waltham, MA, USA) [33].

Serum samples were analyzed using the HITACHI 7170A biochemistry analyzer (Tokyo, Japan) for quantification of triglycerides (TG), total protein (TP), aspartate transaminase (AST), glucose (GLU), alanine transaminase (ALT), and cholesterol (CHOL). Serum ammonia levels were quantified using a commercially available assay kit (no. A086-1-1, Nanjing Jiancheng Biology Engineering Institute, China) following the protocol provided by the manufacturer.

### 2.5. Digestive Physiology

The mid-intestine was homogenized with a solution of 0.86% physiological saline in a volume ratio of 9:1, and the homogenate was centrifuged at 1000× *g* for 10 min at 4 °C. The supernatant was further centrifuged and stored at −80 °C for future analysis. Amylase (AMS) and lipase (LPS) activities were determined using commercially available kits from Nanjing Jiancheng Bioengineering Institute (Nanjing, China), following the manufacturer’s instructions. AMS activity was assessed by measuring the amount of hydrolyzed starch (no. C016-1-1). LPS activity was measured at 580 nm using kit no. A054-2-1. Protease (PRT) activity was determined using the Folin-phenol Ciocalteu’s reagent technique [36]. The mid-intestinal protein content was determined through the application of the Coomassie brilliant blue protein assay method. A microplate reader (Synergy 2, BoiTek, Minneapolis, MN, USA) was used for all absorbance measurements.

Liver segments were processed, embedded in standard paraffin, and sliced into 6-µm-thick sections using a microtome (HistoCore MULTICUT, Leica, Wetzlar, Germany). The sections were subjected to deparaffinization, hydration, staining with hematoxylin and eosin, and then mounting with neutral resin. A microscope (IX51, Olympus, Tokyo, Japan) was used to examine a total of 18 liver sections from each group.

### 2.6. RNA Isolation and Sequencing

Livers from three sturgeons per tank were homogenized in liquid nitrogen and mixed as one transcriptome sample for analysis. Total RNA was extracted according to the protocol of the Trizol reagent kit (Invitrogen, Thermo Fisher Scientific, Waltham, MA, USA). The qualified RNA sample was treated with DNase I, and magnetic beads with Oligo (dT) attached were used to enrich mRNA. The first strand of cDNA was then synthesized with random primers. Subsequently, RNaseH, deoxyribonucleoside triphosphates (dNTPs), DNA polymerase I, and buffer were added to synthesize the second strand of cDNA. After purification and screening, PCR amplification was conducted to construct a cDNA library. The sequencing of a qualified cDNA library was conducted on an Illumina HiSeq 2500 (Illumina Inc., San Diego, CA, USA) for data analysis.

### 2.7. Gene Function Annotation

After removing the adapter sequences and low-quality reads, high-quality clean data were acquired. The Trinity software was used to assemble and splice the sequences to obtain the transcript sequences [37]. To compare the assembled unigenes sequences with the Gene Ontology (GO) [38], Cluster of Orthologous Groups of Proteins (COG) [39], and Kyoto Encyclopedia of Genes and Genomes (KEGG) [40] databases by the BLAST software. The predicted amino acid sequences of unigenes were evaluated using the HMMER 3.1 software, and the results were compared with the entries of the Protein Family (Pfam) database to obtain annotation information on the corresponding gene functions [41].

### 2.8. Differentially Expressed Genes (DEGs) Analysis

After sequence alignment, the annotation file of the reference genome was used to calculate the fragments per kilobase of transcript per million mapped read values (FPKM) value of each transcript in the samples, which was used as the expression level of the transcript [42]. The higher the transcript abundance, the higher the gene expression level. By performing sample-to-sample differential significance analysis of the expression level of each transcript, the DEGs were obtained. The significance was judged by the false discovery rate (FDR) ≤ 0.01 and Fold Change ≥ 2.

### 2.9. Quantitative Real-Time Polymerase Chain Reaction (qRT-PCR)

To validate the transcriptome library screening, qRT-PCR was used to assess the expression of eight genes associated with metabolism. Primers (Table 2) were designed using the Premier 5.0 software according to the transcriptome sequences and synthesized by Shanghai Genechem Co., Ltd (Shanghai, China). The qRT-PCR assays were performed using SYBR Green PCR Master Mix (Takara, Dalian, China) on a Bio-Rad CFX Connect Real-Time System (Bio-Rad Inc., Hercules, CA, USA). The melting curve analysis was performed with conditions ranging from 65 °C to 95 °C for 30 s, and the fluorescence was recorded. All qRT-PCR was repeated three times. The relative expression of the target was calculated using the 2^−ΔΔCt^ method, with β-actin serving as an endogenous reference [43].

### 2.10. Calculations and Statistical Analysis

WGR, FCR, and CF were calculated via the following formulas:

WGR = 100 × [(final weight−starting weight)/starting weight].

CF = body weight (g) × 100/body length^3^ (cm).

FCR = feed intake (g)/weight gain (g).

The apparent digestibility (ADC) of the dry matter, gross energy, crude protein, and crude lipid in the diet was calculated using the following equation: ADC of nutrient or energy (%) = 100 × (1 − (dietary Y_2_O_3_/fecal Y_2_O_3_) × (fecal nutrient or energy/dietary nutrient or energy).

The Shapiro-Wilk and Levene’s tests were used to evaluate the normality and homogeneity assumptions of these data, respectively. A paired Student’s *t*-test and a Wilcoxon signed rank test were applied to compare these data with normal and non-normal distributions, respectively. Statistical significance was regarded at a *p*-value < 0.05, and statistical analysis was conducted using SPSS 23.0 (SPSS Inc., Chicago, IL, USA). Column graphs were generated by GraphPad Prism 9.0 (GraphPad Software, San Diego, CA, USA).

## 3. Results

### 3.1. Growth

No mortality was observed throughout the 56-day trial period. The substitution of fishmeal with CPC significantly reduced WGR (*p* < 0.05), but it was accompanied by a significant increase in FCR (*p* < 0.05) (Table 3).

### 3.2. Body Composition

Replacing fishmeal with CPC decreased the contents of crude lipids and protein in the whole body (*p* < 0.05) while increasing ash content (*p* < 0.05) (Table 4). Dietary CPC caused a significant decrease in the levels of EAAs such as phenylalanine, lysine, valine, isoleucine, arginine, and total amino acid contents (*p* < 0.05), but histidine showed an opposite trend (*p* < 0.05) (Table 5). Non-essential amino acids (NEAAs) such as glycine, alanine, tyrosine, and proline decreased significantly (*p* < 0.05), while serine increased (*p* < 0.05).

### 3.3. ADC of Nutrients

There were significant reductions in ADC of crude lipid, crude protein, dry matter, and gross energy compared to the control group (*p* < 0.05) (Table 6).

### 3.4. Digestive Physiology

Table 7 presents the results of the mid-intestinal enzyme activity of *A. schrenckii*. PRT, LPS, and AMS activities were significantly lower in the CPC group (*p* < 0.05).

**Figure 1:**
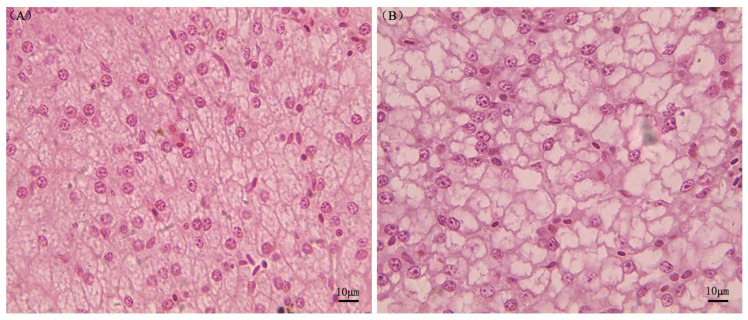
Photomicrographs of histological sections of *A. schrenckii* livers. Note: Panel (**A**) displays hepatocytes with proper nuclei, while panel (**B**) shows fatty infiltration of hepatocytes. The sections were stained with hematoxylin and eosin.

Liver histology showed normal results in individuals fed the fishmeal diet, with abundant granular and eosinophilic cytoplasm, central nuclei, and large nucleoli. Little lipid deposits were present (Figure 1A). Sturgeons fed CPC-containing diets, on the other hand, showed fatty infiltration of hepatocytes and nuclei peripheral displacement in hepatocytes (Figure 1B).

### 3.5. Hematological Parameters

Table 8 showed that dietary CPC had a significant increment in the activity of AST and ALT, as well as the concentration of ammonia in serum (*p* < 0.05). By contrast, significantly decreased levels of TG were observed in the CPC group (*p* < 0.05). No significant differences in the concentrations of TP, GLU, and CHOL were detected between the two groups (*p* > 0.05).

### 3.6. Transcriptomic Sequencing

To obtain quality metrics of the raw reads, we performed quality control checks on the sequencing data using FastQC (v.0.11.4) software. The quality of the sequencing data was assessed, with an average of 4.2 GB of clean data obtained per sample and a Q30 base percentage of over 85%. Raw data were deposited in the NCBI Sequence Read Archive (SRA) database under BioProject number PRJNA736603.

### 3.7. Gene Functional Annotation and Categorization

**Figure 2:**
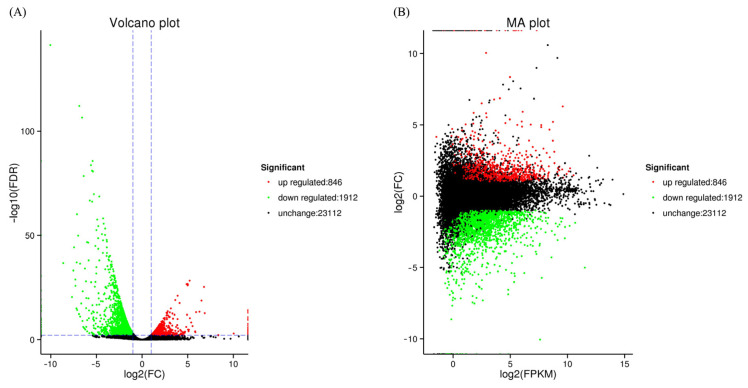
Differentially expressed genes in treatment and control groups. Note: Panels (**A**,**B**) display the comparison of the two groups using a volcano plot and a MA plot, respectively. Red dots represent genes that are strongly up-regulated, green dots represent significantly down-regulated genes, and the absence of black dots indicates that the gene expression was unaffected.

A volcano plot was constructed (Figure 2A) as well as an M-versus-A (MA) plot (Figure 2B) to illustrate the general trend of gene expression levels and comparative multiples among samples. A total of 2758 DEGs were identified, with 846 up-regulated and 1912 down-regulated in the treatment group.

**Figure 3:**
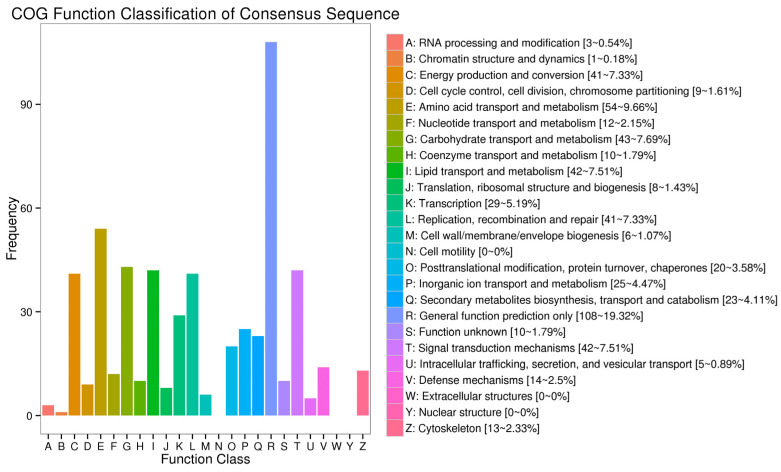
Gene ontology (GO) categorization of DEGs in the treatment and control groups of unigenes.

GO functional analysis classified 902 DEGs into three categories (Figure 3). The top three subcategories of the biological process were the “cellular process”, “metabolic process”, and “single-organism process”. For the cellular component category, the major subcategories were “cell portion”, “organelle”, and “membrane part”. At the same time, “binding activity”, “catalytic activity”, and “transporter” were the most ones in the molecular function category.

### 3.8. Analysis of DEGs

**Figure 4:**
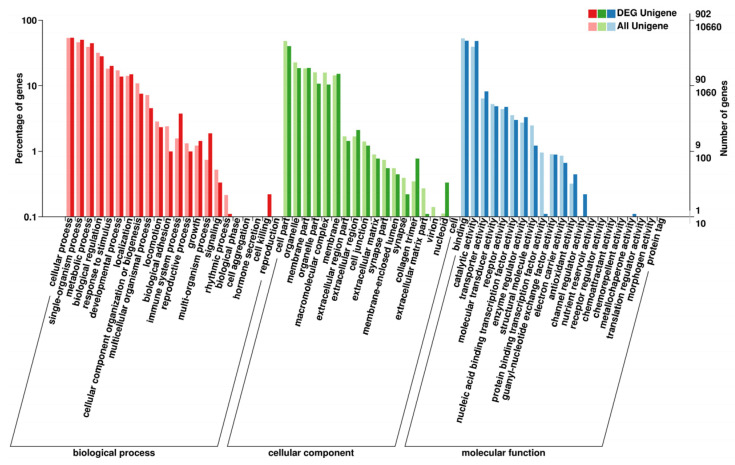
COG function categorization of *A. schrenckii* transcriptome unigenes.

According to the COG function classification of the unigenes, the most enriched group was “general function prediction only” (19.32%), followed by “amino acid transport and metabolism” (9.66%), “carbohydrate transport and metabolism” (7.69%), “signal transduction mechanisms” (7.51%), “replication, recombination, and repair” (7.33%), “energy production and conversion” (7.33%), “lipid transport and metabolism” (7.51%), “transcription” (5.19%), and “inorganic ion transport and metabolism” (4.47%) (Figure 4).

**Figure 5:**
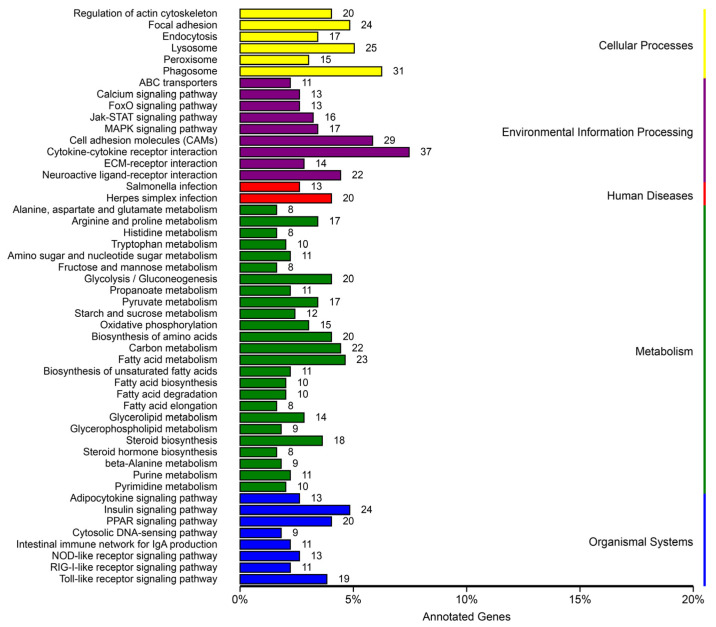
Kyoto encyclopedia of genes and genomes (KEGG) pathway categorization of DEGs with a corrected *p*-value of 0.05 in the treatment and control group comparisons.

These TOP 50 pathways were observed, including metabolism (25 pathways), organismal systems (8 pathways), diseases (2 pathways), environmental information processing (9 pathways), and cellular processes (6 pathways) based on the KEGG analysis (Figure 5). 

**Figure 6:**
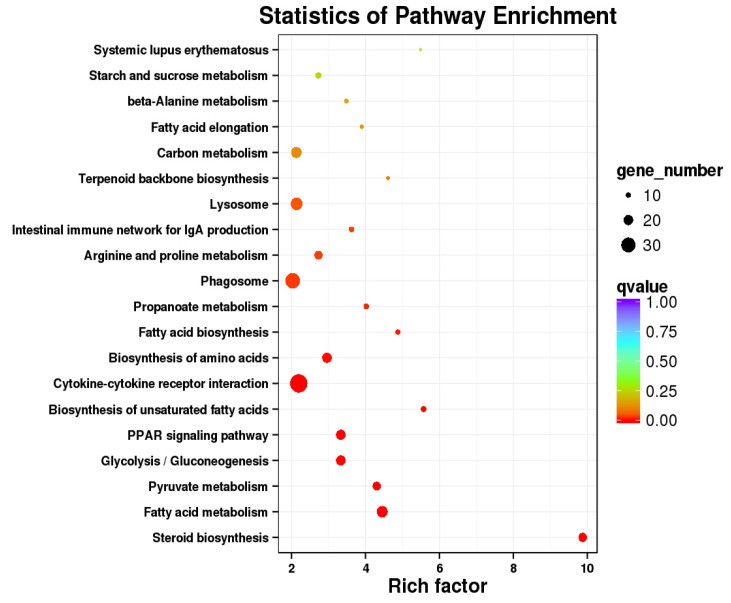
Scatterplot of the top 20 most enriched *A. schrenckii* KEGG pathways. Note: The abscissa shows the enriched element in each pathway, and the ordinate represents the number of routes.

Subsequently, pathway enrichment analysis was performed, and the top 20 significantly enriched pathways were identified, including steroid biosynthesis, fatty acid metabolism, pyruvate metabolism, propanoate metabolism, cytokine-cytokine receptor interaction, biosynthesis of amino acids, fatty acid biosynthesis, glycolysis/gluconeogenesis, biosynthesis of unsaturated fatty acids, PPAR signaling pathway, and other pathways (Figure 6).

### 3.9. Gene Expression Profiling

The top 100 DEGs in the liver transcriptome of sturgeons fed CPC diets were identified through gene expression profiling analysis (Table S1). The genes discovered were linked to a range of metabolic pathways, including secondary metabolites biosynthesis, transport, and catabolism, carbohydrate transport and metabolism, amino acid transport and metabolism, nucleotide transport, lipid transport and metabolism, inorganic ion transport and metabolism, coenzyme transport and metabolism. Furthermore, they were connected with various biological processes, including chromatin structure and dynamics, transcription, chromosome partitioning, cell division, cell cycle control, extracellular structures, defense mechanisms, vesicular transport, secretion, intracellular trafficking, signal transduction mechanisms, energy production and conversion, chaperones, protein turnover, and post-translational modification.

This study focused on the DEGs related to metabolic pathways. Genes related to amino acid transport and metabolism, such as elastase-1 (EL1) and excitatory amino acid transporter 1 (EAAT1), were generally upregulated by diets containing the CPC ingredient. Conversely, transcripts for argininosuccinate synthase (ASS1), cytosolic carboxypeptidase 2 (CCP2), y^+^ L amino acid transporter 2 (y^+^ LAT2), and mast cell protease 1A-like (MCP-1) showed downregulation. Similarly, fructose-1,6-bisphosphatase (FBPASE) was downregulated, while alpha-enolase (ENO1) was upregulated. Concerning lipid transport and metabolism, liver polyprenol reductase (SRD5A3) was downregulated, while most other genes in this category were upregulated, including fatty acid desaturase 1 (FADS1), fatty acid desaturase 2 (FADS2), fatty acid-binding protein 7 (FABP7), fatty acid-binding protein 2 (FABP2), fatty acid-binding protein 1 (FABP1), fatty acid-binding protein 10-A (FABP10A), elongation of very long chain fatty acids protein 6 (ELOVL6), 3-hydroxy-3-methylglutaryl-coenzyme A reductase (HMGCR), acyl-CoA desaturase-like (ADS), elongation of very long chain fatty acids protein 5 (ELOVL5), diphosphomevalonate decarboxylase (MVD), and fatty acid-binding protein 3 (FABP3). In secondary metabolite biosynthesis, transport and catabolism, and cytochrome P450 3A27 (CYP3A27) were downregulated, while ATP-binding cassette sub-family B member 8 (ABCB8), cholesterol side-chain cleavage enzyme, mitochondrial (Precursor) (CYP11A1), and bile salt export pump-like (BSEP) were upregulated. In nucleotide transport and metabolism, inorganic ion transport metabolism, and coenzyme transport and metabolism, adenylosuccinate synthetase isozyme 1 B (ADSSL1) and farnesyl pyrophosphate synthase (FDPS) were upregulated, while solute carrier family 26 member 9 (SLC26A9) was downregulated when sturgeons were fed the CPC diet.

### 3.10. Quantification of qRT-PCR

**Figure 7:**
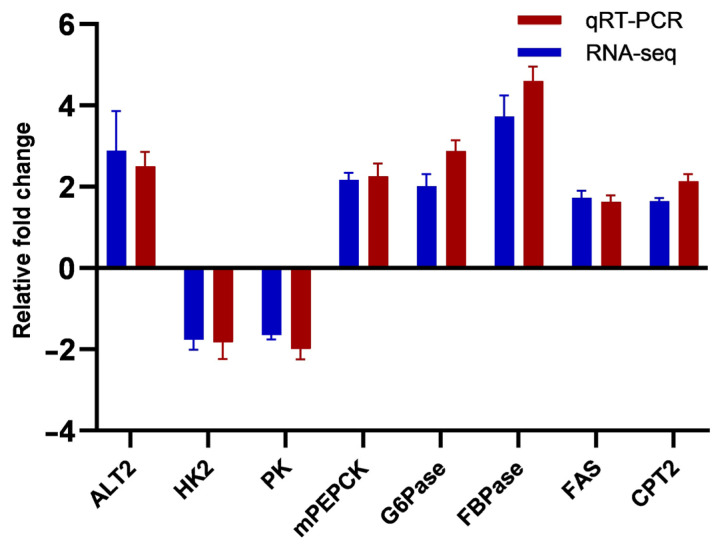
RNA-Seq validation by quantitative PCR (qPCR). Note: It was conducted to confirm the repeatability of the RNA-Seq pipeline’s differential expression data. These data are reported as means ± error (*n* = 3).

The current research verified the DEGs, which were identified with the aid of RNA-Seq, using qRT-PCR of eight crucial metabolic enzymes (Figure 7). The results of the RNA-Seq and qRT-PCR assays for the eight DEGs revealed an up-regulation pattern of alanine aminotransferase 2 (ALT2), phosphoenolpyruvate carboxykinase mitochondrial isoform (mPEPCK), glucose 6-phosphatase (G6Pase), fructose 1,6-bisphosphatase (FBPase), fatty acid synthase (FAS), and carnitine palmitoyltransferase 2 (CPT2), and a down-regulation pattern of hexokinase (HK2) and pyruvate kinase (PK).

## 4. Discussion

### 4.1. Nutritional Physiology

Previous research has demonstrated that replacing fishmeal in aquatic animal feed with plant protein, terrestrial animal protein, or single-cell protein can slow down the growth of fish and reduce feed efficiency [44]. The recommended range for replacing fishmeal with CPC varies greatly among fish species. For example, the growth of hybrid grouper (♀*Epinephelus fuscoguttatus* × ♂*Epinephelus lanceolatu*) [45] or big yellow croaker (*Larimichthys crocea*) [46] was not affected by dietary levels of up to 60% CPC in the feed. The optimum replacement level with CPC in *T. ovatus* was 25.93% [12]. Fishmeal replaced with a plant protein mix (18% wheat gluten meal, 40.8% CPC, and 23% soy protein concentrate) in the feed caused a disturbance in the spiral valve intestinal microbiota and increased morbidity with liver illness in the sturgeon [47]. In this research, the WGR of *A. schrenckii* in the fishmeal-free group (CPC group) was 12.37% lower, and the FCR was 12.84% higher compared to the fishmeal group. The differences in results may be attributed to differences in CPC substitution levels, gossypol content, amino acids, and species [48]. Furthermore, partial substitution at appropriate levels may lead to enhanced benefits at lower costs. Additional studies are needed to determine the optimal level of fishmeal replacement with CPC for *A. schrenckii* aquaculture. Comparing the effects of partial and complete substitution will also provide insights into their mechanisms of action.

Similar to this study, Wu et al. (2022) also found that the level of blood ammonia, a vital product of amino acid catabolism, increased in sturgeons fed with a CPC diet due to unbalanced dietary amino acid compositions and decreased amino acid uptake [49]. The mid-intestinal digestive enzyme activities of *A. schrenckii* decreased when dietary fishmeal was completely replaced with CPC. The same trend was also reported in hybrid groupers [50]. Li and Robinson (2006) reported that gossypol, present in cottonseed meals, can adversely affect the development and digestive function of fish [51]. Gossypol has been shown to interact with ε-amino acids and is typically excreted from the body. In addition, it can connect to enzymes such as pepsinogen and trypsin in the digestive tract, obstructing their activities [52]. The gossypol content in this study was 305.12 mg/kg, which may interfere with intestinal digestion and absorption. Tian et al. (2022) demonstrated that high dietary concentrations of CPC (75%) had detrimental effects on the growth performance and feed utilization of *L. crocea*, likely due to their influence on intestinal digestion and absorption [46]. Therefore, it is critical to decreasing the gossypol content of CPC through techniques such as raw material processing to improve its utilization ratio.

Consumption of cottonseed gossypol may result in liver damage and associated serum biochemical alterations [53]. Rinchard et al. (2003) suggested that the liver is a key organ for the metabolism of gossypol [54]. Subsequently, liver damage can reduce glycogen dissociation and synthesis, resulting in decreased serum GLU concentrations [55]. In the present study, *A. schrenckii* eating a diet comprising 50% CPC showed significantly lower serum GLU, TG, and TP concentrations, which may be due to either a lack of nutrition in the diet or alterations in the metabolism of GLU and proteins in the liver [56]. The reduction in serum TG concentration suggests that TG can be used as a substitute for GLU to provide nourishment and energy [57]. The administration of gossypol to rats has been shown to increase serum ALT and AST activity [58]. Similarly, in *A. schrenckii* fed a diet containing 100% CPC, serum transaminase activity increased significantly, and there was visible structural damage to the liver. These changes in biochemical parameters were likely due to liver damage or malnutrition, which could be attributed to toxic chemicals in great abundances, such as free gossypol or other antinutrients [58].

In this study, total amino acid levels in *A. schrenckii* were significantly lower when fed a diet consisting of 50% CPC. CPC may contain higher levels of gossypol and condensed tannins that may have influenced amino acid digestibility [59]. Dietary CPC resulted in a decrease in EAAs such as phenylalanine, lysine, valine, isoleucine, and arginine (*p* < 0.05), while NEAAs such as glycine, alanine, tyrosine, and proline increased significantly (*p* < 0.05). This showed that replacing fishmeal with CPC completely changes the body composition profile and may result in lower flesh quality in *A. schrenckii*, a decreased amount of EAAs, and flavor-enhancing amino acids (glycine and alanine). 

### 4.2. Liver Transcripts

Up to now, the influence of substituting fishmeal with alternative protein sources on the transcriptome has not been well studied. In this experiment, the GO and KEGG annotation classification results of the hepatic transcriptome analysis of *A. schrenckii* indicated that the CPC diet caused greater DEGs involved in metabolic processes such as “carbohydrate transport and metabolism” (7.69%), “amino acid transport and metabolism” (9.66%), and “lipid transport and metabolism” (7.51%). The study also found that the CPC diet reduced the growth performance and nutrient deposition in the body of *A. schrenckii*, which impacted lipid transport and metabolism, amino acid transport and metabolism, carbohydrate transport and metabolism, and energy production and conversion in the liver. Similar findings have been reported in *S. salar* [60], European seabass (*Dicentrarchus labrax*) [61], and *O. mykiss* [62], where the expression of genes associated with primary metabolic functions was modified by dietary components.

#### 4.2.1. Protein Synthesis

In this study, the hepatic amino acid synthesis pathway of *A. schrenckii* was changed when fishmeal in the diet was fully replaced by CPC. This can be attributed to the high protein requirement of *A. schrenckii* and its increased sensitivity to changes in dietary protein quality [63]. The amino acids in diets, specifically fishmeal, may stimulate protein synthesis via the amino acid receptor signaling pathway and the mTOR signaling pathway [64]. The gene expression value of mTOR did not show a significant difference in this study, but the biosynthesis of amino acids was the most significantly enriched route linked to protein synthesis. The findings are consistent with a previous study on Japanese yellowtail (*Seriola quinqueradiata*) that found lower expression levels of digestive enzyme genes in a diet that replaced fishmeal with soybean meal and maize gluten meal [65]. Interestingly, the expression level of genes (i.e., EL1, CCP2, and MCP-1) in the liver of sturgeons fed the CPC diet was found to be lower than that of the control diet, which is consistent with lower activity of digestive enzymes in the mid-intestine and indicates a hindrance in their digestive ability. In this study, RNA-Seq data showed that genes involved in amino acid synthesis and transport, such as ASS1 and y^+^ LAT2, were suppressed by the CPC substitution of fishmeal. LAT2 may also serve as the primary large neutral amino acid transporter in the liver [66]. When fish were fed a plant protein mix diet, it significantly increased the gene expression of L-type AA transporters (LAT2) and asparagine synthetase enzymes in *S. maximus* [67]. Therefore, these may result in reduced EAAs content and potentially decreased fish protein synthesis.

#### 4.2.2. Lipid Metabolism

In the present study, the pathways found to be significantly impacted in the CPC group were steroid biosynthesis, fatty acid metabolism, fatty acid synthesis, unsaturated fatty acid synthesis, and the PPAR signaling pathway (Figure 6). Other studies have reported that the regulation of lipid metabolism and transport in fish can be influenced by the use of alternative protein sources instead of fishmeal in feed [26,68]. There was a significant increase in the expression of genes related to cholesterol synthesis and fatty acid desaturation, including MVD and HMGCR, in the CPC group. Fishmeal is the main source of steroids in aquafeeds, and when it is completely replaced by vegetable protein, steroids are absent. The MVD, a key enzyme in the mevalonate pathway, is involved in transferring cholesterol and lipids from the liver to other tissues [69]. HMGCR, the enzyme that catalyzes the de novo synthesis of cholesterol, directly impacts the rate of cholesterol synthesis and the cholesterol concentration in the body [70]. In the present research, a significant increase in the mRNA expression of the HMGCR gene was seen in the CPC group compared to the control, indicating an increased ability to synthesize endogenous cholesterol. However, hepatic cholesterol accumulation was found in the CPC group, and hematoxylin and eosin staining revealed fatty infiltration of hepatocytes (Figure 1). As a result, the CPC group of sturgeons may satisfy their physiological needs by increasing the synthesis of endogenous steroids, implying an increased demand for steroids in steroid-free feed for sturgeons, consistent with previous studies on *S. salar* [71].

In this study, the lipid metabolism and transport genes, including FADS1, FADS2, ELOVL5, ELOVL6, FABP2, FABP3, FABP7, and FABP10A in the *A. schrenckii* liver, showed significant upregulation in the CPC group. These genes play a critical role in the synthesis, transport, and metabolism of fatty acids. FABP2, FABP3, FABP7, and FABP10A are fatty acid-binding proteins involved in intracellular transport and fatty acid metabolism regulation. FADS1 and FADS2 are involved in the biosynthesis of n-3 and n-6 polyunsaturated fatty acids (PUFAs) and their subsequent metabolisms, such as anti-inflammatory effects, signal transduction, and cell membrane structure. ELOVL5 and ELOVL6 are genes related to the extension of long-chain fatty acids (LCUFA). The increased expression of these genes indicates that when cottonseed protein is used to replace fishmeal in the diet, it promotes changes in liver fatty acid synthesis, transport, and metabolism to meet the energy needs of sturgeons. Similar studies [26,68] have shown that alternative protein sources in feed can regulate lipid metabolism and transport in fish. When cottonseed protein was used to replace fishmeal in the diet, higher levels of FADS1 and FADS2 were observed. This is attributed to the substitution of fishmeal (containing 8% lipid and abundant in n-3 LCUFA) by plant protein (deficient in LCUFA). When fishmeal is replaced, FADS1 and FADS2 are involved in synthesizing LCUFA [72,73]. Therefore, when fishmeal is fully replaced by cottonseed protein in the diet, sturgeons maintain their physiological processes by regulating the synthesis, transport, and metabolism of fatty acids.

#### 4.2.3. Carbohydrate Metabolism

Replacing fishmeal with corn protein concentrate (CPC) in the feed significantly affected the carbohydrate metabolism of *A. schrenckii*. Results from transcriptomic DEGs and KEGG pathway enrichment analysis showed a significant correlation with three carbohydrate metabolism pathways: pyruvate metabolism, glycolysis, and gluconeogenesis (Figure 6). Pyruvate metabolism is the first step in glucose oxidation, which occurs when glucose is converted to ketone salts. Then, ketone salts undergo glycolysis, producing energy as ATP [74]. Glucose can be synthesized from non-carbohydrate precursors such as amino acids and fatty acids, which help fish maintain their glucose levels. The essential amino acid proportion and balance of CPC are not as good as fishmeal, which may impact the protein synthesis of sturgeon and thus alter pyruvate metabolism, glycolysis, and gluconeogenesis. Additionally, dietary CPC affects the lipid metabolism of sturgeon, which in turn affects glucose uptake and storage, leading to changes in glycolysis and gluconeogenesis.

The gene expression profiles of multiple enzymes implicated in carbohydrate metabolism changed in *A. schrenckii* fed a 50% CPC diet. Specifically, HK2 decreased 1.76-fold, PK decreased 1.64-fold, mPEPCK increased 2.17-fold, G6Pase increased 2.01-fold, FBPase increased 3.51-fold, and ENO1 increased 4.18-fold. These changes suggest that replacing fishmeal with CPC may impact glucose metabolism in *A. schrenckii*. The decrease in HK2 and PK enzymes may indicate a reduction in glucose synthesis and carbohydrate metabolism activities, respectively [75]. This could be due to differences in the types of protein and other nutrients in fishmeal and CPC, as well as their bioavailability to fish [76]. Conversely, the increase in mPEPCK and G6Pase enzymes suggests an increase in carbohydrate synthesis and excretion, which could be a way for the fish to maintain glucose homeostasis as plant protein may not be as efficient at supplying energy as fishmeal [8,77]. The increases in FBPase and ENO1 may also suggest an increase in the synthesis of fructose-6-phosphate and pyruvate, which could provide more energy to fish [78]. The increased expression of ENO1, an enzyme involved in fermentation, observed in fish fed with plant protein-based diets may suggest that fermentation is more extensively used as a means of energy production due to the lower energy efficiency of plant protein compared to fishmeal [79]. These changes indicate a shift in the metabolic pathways used by the liver to generate energy, potentially having negative effects on the development and health of *A. schrenckii* fed a CPC-based diet.

## 5. Conclusions

In conclusion, *A. schrenckii* fed a fishmeal-free diet containing 50% CPC exhibited lower growth and feed efficiency, as well as lower levels of EAAs and higher levels of NEAAs. Mid-intestinal enzyme activity was reduced, and liver tissue histology revealed fatty infiltration of hepatocytes. Gene expression analysis revealed several metabolic pathways, including those for the metabolism of amino acids, lipids, and glucose. These results suggest that completely replacing fishmeal with CPC may be adverse to *A. schrenckii*. This study provides critical information for sturgeon aquaculture, such as developing innovative aquafeeds and evaluating diet performance using molecular approaches.

## Figures and Tables

**Table 1:** Nutrients and ingredients used to prepare the diets (air-dry basis, %).

Ingredients	Groups	
	Control	Treatment
Fishmeal ^1^	50.00	—
Cottonseed protein concentrate ^2^	—	50.00
Soybean meal ^3^	22.00	22.00
Wheat middlings ^4^	19.75	16.15
Fish oil ^5^	5.00	7.50
Soy lecithin ^6^	2.00	2.00
Ca(H_2_PO_4_)_2_ ^7^	—	1.00
Betaine ^8^	—	0.10
Butylated hydroxytoluene ^9^	0.05	0.05
Choline chloride ^10^	0.20	0.20
Vitamin premix ^11^	0.30	0.30
Mineral premix ^12^	0.60	0.60
Arginine ^13^	1.88	—
Lysine ^14^	—	1.10
Methionine ^15^	—	0.08
Yttrium oxide ^16^	0.10	0.10
Nutritional level ^17^		
Moisture	9.63	9.51
Crude protein	44.55	44.24
Crude lipid	10.45	10.53
Ash	6.88	6.40
Gross energy (MJ/kg)	19.21	19.88
Gossypol (mg/kg)	0.00	305.12
Arginine	4.69	4.71
Histidine	1.20	1.32
Isoleucine	1.87	1.60
Leucine	3.41	2.86
Lysine	3.29	3.26
Methionine	1.05	1.02
Threonine	1.89	1.46
Tryptophan	0.55	0.15
Phenylalanine	1.93	2.43

Note: ^1−10^ Weierhao Feed Ingredients Corporation, Harbin, China. ^11^ Vitamin premix (mg/kg): nicotinic acid 275, thiamin 25, retinol acetate 5.2, folic acid 8, riboflavin 40, cyanocobalamin 0.8, cholecalciferol 0.07, ascorbic acid 200, biotin 5, pantothenic acid 100, pyridoxine 25, α-tocopherol 100, and menadione sodium bisulfate 5. ^12^ Mineral premix (mg/kg): magnesium sulfate heptahydrate 2000, potassium chloride 1500, iron sulfate heptahydrate 1000, copper sulfate pentahydrate 20, manganese sulfate tetrahydrate 100, zinc sulfate heptahydrate 150, potassium iodide 3, sodium chloride 500, cobalt chloride 5, and sodium selenite 3. ^13−16^ Sigma-Aldrich, St. Louis, MO, USA. ^17^ Nutrient levels were determined values.

**Table 2:** Primers used for quantitative real-time PCR.

Genes	Forward Primer Sequences (5′–3′)	Reverse Primer Sequences (5′–3′)	PCR Fragment Length (bp)	Accession Number
ALT2	GGGAGGTGCTTGGACGTAAA	GGGATAGCTCTACACACCGC	194	XM_032070390.1
PK	GCAGGTTCTGGCAGCTAGAA	AGACCCCCACTCCTTTCACT	117	XM_034048533.2
HK2	ATGCACTGAAGGGTTCTGGG	TCATCGCTGGCAGGATGATC	109	XM_034912516.1
FAS	GGAGAAAGCCGTGACTGTGA	GATGTCATAGTCGCGCTCCA	184	XR_004547729.2
CPT2	CTCGAAATTGATGGCAGCCG	CTCTTCTGCCTGTGCTTGGA	150	XM_041270617.1
FBPase	AGCAGTTGACTCCTTGTCCG	AGCCCACAGAGAAAGATGCC	114	XM_034902448.1
G6Pase	CTACATGGAGAGCTGCCGAG	AGGAATGATGGCCACAGTGG	190	XM_034055351.2
mPEPCK	TGCTGAGTCTGGTTCTGCTG	GCCTGACCAGCAACCCTTAT	112	XM_034920005.1
β-actin ^1^	GGTTTCGCTGGAGATG	ATACTTCAGGGTCAGGATA	150	XM_034412759.1

Note: ^1^ As an internal reference, the *A. schrenckii* β-actin gene was used as a housekeeping gene. ALT2, alanine aminotransferase 2; HK2, hexokinase; PK, pyruvate kinase; mPEPCK, phosphoenolpyruvate carboxykinase mitochondrial isoform; G6Pase, glucose 6-phosphatase; FBPase, fructose 1,6-bisphosphatase; FAS, fatty acid synthase; CPT2, carnitine palmitoyltransferase 2.

**Table 3:** Comparison of growth performance in *A. schrenckii*.

	Control	Treatment
IBW/g	21.61 ± 0.50	21.53 ± 0.56
FBW/g	93.23 ± 2.47 *	84.04 ± 3.84 *
WGR/%	331.79 ± 20.88 *	290.76 ± 26.66 *
FCR	1.09 ± 0.01 *	1.23 ± 0.03 *
CF	0.38 ± 0.01 *	0.33 ± 0.02 *

Note: Values are represented as the mean ± S.E. of pooled data from triplicates per treatment (*n* = 3). Means in the same row with asterisk subscripts are significantly different (*p* < 0.05). IBW, initial body weight; FBW, final body weight; WGR, weight gain rate; FCR, feed conversion ratio; CF, condition factor.

**Table 4:** Comparison of whole-body composition in *A. schrenckii*.

Groups	Moisture (%)	Crude Protein (%)	Crude Lipid (%)	Ash (%)
Control	77.65 ± 0.18	13.63 ± 0.20 *	5.64 ± 0.06 *	3.04 ± 0.23 *
Treatment	77.50 ± 0.36	13.23 ± 0.10 *	5.34 ± 0.05 *	3.88 ± 0.14 *

Note: Values are represented as the mean ± S.E. of pooled data from triplicates per treatment (*n* = 3). Means in the same column with an asterisk subscript are significantly different (*p* < 0.05).

**Table 5:** Comparison of the whole-body amino acid profile in *A. schrenckii*.

Amino Acids	Control	Treatment
Aspartic acid	3.82 ± 0.06	3.58 ± 0.10
Threonine	2.73 ± 0.00	2.69 ± 0.02
Serine	3.15 ± 0.05 *	3.51 ± 0.06 *
Glutamic acid	11.39 ± 0.23	11.04 ± 0.06
Glycine	5.09 ± 0.03 *	4.88 ± 0.04 *
Alanine	4.24 ± 0.03 *	4.15 ± 0.02 *
Cysteine	0.80 ± 0.03	0.87 ± 0.00
Valine	3.28 ± 0.03 *	3.08 ± 0.06 *
Methionine	1.94 ± 0.05	1.81 ± 0.01
Isoleucine	3.12 ± 0.03 *	3.01 ± 0.01 *
Leucine	5.94 ± 0.03	5.87 ± 0.06
Tyrosine	2.44 ± 0.04 *	2.04 ± 0.00 *
Phenylalanine	2.76 ± 0.04 *	2.40 ± 0.03 *
Lysine	6.07 ± 0.03 *	5.64 ± 0.07 *
Histidine	1.26 ± 0.01 *	1.40 ± 0.02 *
Arginine	4.48 ± 0.05 *	4.22 ± 0.04 *
Proline	2.06 ± 0.00 *	1.88 ± 0.03 *
Tryptophan	0.06 ± 0.01	0.05 ± 0.01
Total	64.62 ± 0.29 *	62.25 ± 0.31 *

Note: Values are represented as the mean ± S.E. of pooled data from triplicates per treatment (*n* = 3). Means in the same row with an asterisk subscript are significantly different (*p* < 0.05).

**Table 6:** Comparison of apparent digestibility in *A. schrenckii*.

Groups	ADC of Dry Matter (%)	ADC of Crude Protein (%)	ADC of Crude Lipid (%)	ADC of Energy (%)
Control	65.24 ± 0.53 *	91.11 ± 0.47 *	98.39 ± 0.13 *	89.59 ± 0.30 *
Treatment	46.76 ± 0.70 *	81.77 ± 0.54 *	96.50 ± 0.18 *	62.55 ± 0.66 *

Note: Values are represented as the mean ± S.E. of pooled data from triplicates per treatment (*n* = 3). Means in the same column with an asterisk subscript are significantly different (*p* < 0.05).

**Table 7:** Comparison of mid-intestinal digestive enzymes in *A. schrenckii*.

Indices	Control	Treatment
PRT (U/L)	237.10 ± 6.12 *	146.73 ± 6.92 *
LPS (U/L)	153.19 ± 7.34 *	146.59 ± 6.12 *
AMS (U/L)	159.96 ± 3.18 *	132.31 ± 3.51 *

Note: Values are represented as the mean ± S.E. of pooled data from triplicates per treatment (*n* = 3). Means in the same column with an asterisk subscript are significantly different (*p* < 0.05). PRT, protease; LPS, lipase; AMS, amylase.

**Table 8:** Comparison of serum biochemical indices in *A. schrenckii*.

Indices	Control	Treatment
AST (U/L)	350.00 ± 26.46 *	540.00 ± 55.68 *
ALT (U/L)	15.53 ± 2.96 *	29.93 ± 3.33 *
TP (g/L)	30.73 ± 1.93	29.30 ± 0.82
GLU (mmol/L)	3.89 ± 0.39	3.87 ± 0.27
CHOL (mmol/L)	3.96 ± 0.38	4.20 ± 0.36
TG (mmol/L)	6.24 ± 0.91 *	4.12 ± 0.95 *
Ammonia (μmol/L)	372.33 ± 7.51 *	663.67 ± 19.86 *

Note: Values are represented as the mean ± S.E. of pooled data from triplicates per treatment (*n* = 3). Means in the same row with an asterisk subscript are significantly different (*p* < 0.05). AST, aspartate aminotransferase; ALT, alanine aminotransferase; TP, total protein; GLU, glucose; CHOL, cholesterol; TG, triglycerides.

## Data Availability

The original contributions presented in the study are included in the article, and further inquiries can be directed to the corresponding authors.

## Supplementary Materials

The following supporting information can be downloaded at: https://www.mdpi.com/article/10.3390/biology12040490/s1, Table S1: Liver transcripts related to the top 100 most significant characteristics indicating differential expression between diets.
