# Supplementary material for: Physiological, Nutritional and Transcriptomic Responses of Sturgeon (Acipenser schrenckii) to Complete Substitution of Fishmeal with Cottonseed Protein Concentrate in Aquafeed
Source: Biology (Basel). 2023 Mar 23;12(4):490. doi: 10.3390/biology12040490 (PMC10135981; doi:10.3390/biology12040490)
Supplement: Supplementary file 1 [file biology-12-00490-s001.zip › biology-2270157-supplementary.pdf]

biology MDPI

Table S1. Liver transcripts related to the top 100 most significant characteristics indicating differential expression between diets.

Gene ID	Swiss-Prot description	Gene name	Log$_2$ fold-change
	Amino acid transport and metabolism		
c65665.graph_c0	Argininosuccinate synthase	ASS1	−3.83
c68725.graph_c0	Elastase-1	EL1	3.77
c42948.graph_c0	Cytosolic carboxypeptidase 2	CCP2	−3.67
c60960.graph_c0	y^+L amino acid transporter 2	y^+LAT2	−3.22
c72411.graph_c0	Mast cell protease 1A-like	MCP-1	−3.06
c68540.graph_c0	Excitatory amino acid transporter 1	EAAT1	3.90
	Carbohydrate transport and metabolism		
c55078.graph_c0	Alpha-enolase	ENO1	4.18
c68691.graph_c0	Fructose-1,6-bisphosphatase	FBPASE	3.51
	Lipid transport and metabolism		
c72373.graph_c1	Fatty acid desaturase 2	FADS2	6.43
c72373.graph_c0	Fatty acid desaturase 1	FADS1	5.31
c24778.graph_c0	Fatty acid-binding protein 7	FABP7	5.16
c11825.graph_c0	Fatty acid desaturase 2	FADS2	4.89
c24765.graph_c0	Fatty acid-binding protein 1	FABP1	4.68
c68091.graph_c0	Polyprenol reductase	SRD5A3	−4.28
c52710.graph_c0	Fatty acid-binding protein 2	FABP2	4.05
c81208.graph_c1	Elongation of very long chain fatty acids protein 5	ELOVL5	3.69
c64002.graph_c0	Fatty acid-binding protein 10-A	FABP10A	3.60
c66249.graph_c0	Elongation of very long chain fatty acids protein 6	ELOVL6	3.39
c77228.graph_c0	3-hydroxy-3-methylglutaryl-coenzyme A reductase	HMGCR	3.38
c72798.graph_c0	Elongation of very long chain fatty acids protein 5	ELOVL5	3.29
c77049.graph_c1	Acyl-CoA desaturase-like	ADS	3.27
c70009.graph_c1	Diphosphomevalonate decarboxylase	MVD	3.08
c59830.graph_c0	Fatty acid-binding protein 3	FABP3	3.07
	Nucleotide transport and metabolism		
c64721	Adenylosuccinate synthetase isozyme 1 B	ADSSL1	3.18
	Inorganic ion transport and metabolism		
c79656.graph_c0	Solute carrier family 26 member 9	SLC26A9	−3.15
	Posttranslational modification, protein turnover, chaperones		
c79232.graph_c0	Gastricsin-like	GC	9.76
c79630.graph_c0	Cathepsin E-A (Precursor)	CTSE-A	4.29
c57973.graph_c0	Nuclear factor 7	NF7	−3.83
c76582.graph_c0	Cathepsin E-A (Precursor)	CTSE-E	3.68
c74859.graph_c1	High choriolytic enzyme 1 (Precursor)	HCEA	−3.65
c68767.graph_c1	Cathepsin S (Precursor)	CTSS	−3.51
c74268.graph_c0	Cathepsin K (Precursor)	CTSK	−3.08
c74534.graph_c0	Cathepsin L1 light chain (Fragments)	CTSL	−3.01
	Secondary metabolites biosynthesis, transport and catabolism		
c58469.graph_c0	ATP-binding cassette sub-family B member 8, mitochondrial (Precursor)	ABCB8	3.91
c66490.graph_c0	Cytochrome P450 3A27	CYP3A27	−3.83
c39681.graph_c0	Cholesterol side-chain cleavage enzyme, mitochondrial (Precursor)	CYP11A1	3.74

c79851.graph_c0	Cholesterol side-chain cleavage enzyme, mitochondrial (Precursor; Fragment)	CYP11A1	3.06
c62166.graph_c0	ATP-binding cassette sub-family B member 8, mitochondrial (Precursor)	ABCB8	3.02
c55354.graph_c0	Bile salt export pump-like	BSEP	3.00
	Signal transduction mechanisms		
c64354.graph_c0	Ras-related protein Rab-13 (Precursor)	RAB13	−5.10
c68658.graph_c0	Acetylcholine receptor subunit gamma (Precursor)	CHRNG	4.77
c70004.graph_c0	Serine/threonine-protein kinase H1 homolog	PSKH1	4.35
c62996.graph_c0	Cadherin-1 (Precursor)	CDH1	4.14
c82739.graph_c0	G-protein coupled receptor 126 (Precursor)	GPR126	−3.98
c65831.graph_c0	cAMP-dependent protein kinase type I-alpha regulatory subunit	PRKAR1A	3.68
c69526.graph_c0	Lutropin-choriogonadotropic hormone receptor (Precursor)	LHCGR	3.56
c81491.graph_c0	Semaphorin-3D (Precursor)	SEMA3D	−3.48
c50188.graph_c0	Activin receptor type-2A (Precursor)	ACVR2A	−3.28
c68769.graph_c0	Guanine nucleotide-binding protein subunit alpha-11	GNA11	−3.23
c78283.graph_c0	Receptor-type tyrosine-protein phosphatase U (Precursor)	PTPRU	3.06
c79615.graph_c0	Rho GTPase-activating protein 15	ARHGAP15	−3.04
	Transcription		
c61682.graph_c0	Nascent polypeptide-associated complex subunit alpha	NACA	−4.33
c77567.graph_c1	Transcription factor jun-B	JUNB	−3.30
	Energy production and conversion		
c79486.graph_c0	Nitric oxide synthase, inducible	NOS2	−6.40
c63454.graph_c0	V-type proton ATPase 116 kDa subunit a isoform 1	ATP6V0A1	6.36
c70775.graph_c0	NADH-ubiquinone oxidoreductase chain 5	MT-ND5	−5.90
c53221.graph_c0	NADH-ubiquinone oxidoreductase chain 1	MT-ND1	−5.69
c35297.graph_c0	NADH-ubiquinone oxidoreductase chain 4	MT-ND4	−5.69
c10552.graph_c0	NADH-ubiquinone oxidoreductase chain 3	MT-ND3	−5.59
c6627.graph_c0	ATP synthase subunit alpha, mitochondrial (Precursor)	ATP5A	−4.73
c62909.graph_c0	F1 ATP synthase beta subunit	ATPSB	−4.38
c73793.graph_c0	Acylphosphatase-2	ACYP2	−4.09
c80002.graph_c0	28S ribosomal protein S7, mitochondrial (Precursor)	MRPS7	−4.04
c57962.graph_c0	Retinal dehydrogenase 2	ALDH1A2	−3.98
c68657.graph_c0	Mitochondrial uncoupling protein 2	UCP2	3.96
c77677.graph_c0	SH3 and PX domain-containing protein 2A	SH3PXD2A	−3.74
c57834.graph_c0	Retinal dehydrogenase 2	ALDH1A2	−3.22
c74040.graph_c0	Nitric oxide synthase, endothelial-like	ENOS	−3.00
	General function prediction only		
c61559.graph_c0	Trace amine-associated receptor 13c	TAAR13C	6.17
c71278.graph_c0	Sulfotransferase family cytosolic 1B member 1	SULT1B1	−5.67
c84000.graph_c0	Toll-like receptor 13	TLR13	−5.32
c50413.graph_c0	Substance-P receptor	TACR1	−4.85
c78910.graph_c0	Early growth response protein 2b	EGR2B	−4.36
c62976.graph_c0	Kinase D-interacting substrate of 220 kDa	ANKRD22	−4.09
c62289.graph_c0	Carbonic anhydrase	CAHZ	3.69
c66321.graph_c0	Type-1 angiotensin II receptor A	AGTR1-A	3.58

c62243.graph_c0	Atlastin-2	ATL2	3.54
c78892.graph_c0	Pleckstrin homology domain-containing family F member 2	PLEKHF2	−3.54
c71724.graph_c0	Ras-related protein Rab-13 (Precursor)	RAB13	−3.36
c69066.graph_c0	Radial spoke head 10 homolog B	RSPH10B	−3.32
c70747.graph_c0	Solute carrier family 25 member 47-B	SLC25A47B	−3.26
c72623.graph_c0	Leucine-rich repeat-containing G-protein coupled receptor 4 (Precursor)	LGR4	3.14
c69538.graph_c0	c-C chemokine receptor type 3-like	CCRL3	−3.13
c82039.graph_c0	Neuropeptide Y receptor type 1	NPY1R	2.99
	Intracellular trafficking, secretion, and vesicular transport		
c63522.graph_c0	Ras-related protein Rab-5C	RAB5C	−3.08
	Cell cycle control, cell division, chromosome partitioning		
c38737.graph_c0	Caspase-2 subunit p12 (Precursor)	CASP2	−4.18
c60461.graph_c0	Caspase-14-like	CASP14	−3.78
	Chromatin structure and dynamics		
c78999.graph_c0	Olfactomedin (Precursor)	OLFM4	3.20
	Coenzyme transport and metabolism		
c68949.graph_c0	Farnesyl pyrophosphate synthase	FDPS	3.23
	Cytoskeleton		
c49570.graph_c0	Tektin-4	TEKT4	5.84
c70361.graph_c0	Adseverin	SCIN	−4.29
c79796.graph_c0	Macrophage-capping protein-like	CAPG	-3.04
	Defense mechanisms		
c74811.graph_c0	Dual specificity protein phosphatase 22-A	DUSP22	-3.29
	Extracellular structures		
c59482.graph_c0	16 kDa beta-galactoside-binding lectin	C-16	6.52
c78742.graph_c0	Olfactomedin (Precursor)	OLFM4	4.98
c24982.graph_c0	Collagen alpha-1(I) chain (Precursor)	COL1A1	4.49
c61333.graph_c0	Collagen alpha-1(II) chain (Precursor; Fragment)	COL2A1	3.78
c67365.graph_c0	Collagen alpha-1(II) chain (Precursor)	COL2A1	3.39
c81525.graph_c0	Integrin alpha-8 light chain (Precursor)	ITGA8	−3.39
c57599.graph_c0	Integrin alpha-6 light chain (Precursor)	ITGA6	−3.31
